# Mechanisms of the decrease in low-temperature electrochemical performance of Li_4_Ti_5_O_12_-based anode materials

**DOI:** 10.1038/s41598-017-15504-4

**Published:** 2017-11-10

**Authors:** Qian Huang, Zhen Yang, Jian Mao

**Affiliations:** Sichuan University, College of Materials Science and Engineering, Chengdu, 610064 China

## Abstract

The electrochemical performances of Li_4_Ti_5_O_12_ (LTO) and Li_4_Ti_5_O_12_-rutile TiO_2_ (LTO–RTO) composite electrodes at low temperatures were evaluated. The electrochemical performance of both electrodes decreased at low temperatures; regardless, the LTO–RTO electrode performed better than the LTO electrode. First, high viscosity and low ion conductivity of liquid electrolytes at low temperatures significantly reduce electrochemical performance. Second, cycling at low temperatures changes the crystal structure of LTO–based electrodes, impeding lithium ion diffusion and even causing the diffusion path to change from easy to difficult. However, changes in the crystal structure of the LTO–RTO electrode were not sufficient to change this path; thus, diffusion continued along the 8a-16c-8a pathway. Finally, from the perspective of dynamics, aggravation of a side reaction, increase in charge transfer resistance and polarization, and decrease in lithium ion diffusion at low temperatures reduce the electrochemical performance of LTO–based anode materials. However, the activation energy based on lithium ion diffusion is lower in the LTO–RTO electrode than the LTO electrode. The results confirmed that the electrochemical performance of the LTO–RTO electrode was better than that of the LTO electrode at low temperatures.

## Introduction

Fossil fuel has started to diminish, and air pollution has escalated in severity; thus, clean and renewable energy sources are urgently needed. Li-ion batteries, which exhibit high energy density and non-memory effect and support quick charging, can efficiently replace fossil fuel as energy source for electric vehicles, plug-in hybrid electric vehicles, and energy storage devices^1–3^. Li_4_Ti_5_O_12_ (LTO) is indisputably a high-potential anode material because of its distinct features, including the following: “zero-strain” structure during charge/discharge, which ensures a long cycling life^4,5^; high charge/discharge platform, which can avoid the deposition of lithium metal on the electrode surface and the formation of lithium dendrites during charge/discharge at high rates or low temperatures, thereby strengthening safety^6^; and 2-phase electrochemical reaction mechanism. The last feature indicates that the voltage distinctly changes when a phase completely transforms to another, signaling the end of charging/discharging and thus ensuring safety^6,7^. Low ionic and electron conductivities have long been acknowledged to significantly influence performance; as such, these factors have been largely studied. The discharge specific capacity of the LTO electrode has exceeded 140 mAh g^−1^ at 20 C (1 C = 175 mA g^−1^) by optimization methods, such as decreasing particle size, controlling morphology, doping ions, coating, compositing, and so on^8–12^. However, using the LTO anode material in Li-ion batteries is challenged with a non-negligible problem in its practical application, which is poor electrochemical performance at low temperatures^13^. The low-temperature electrochemical performance of the LTO electrode was first studied by Allen *et al*.^14^. Since then, researches have been conducted on the influence of synthetic methods, carbon coating, La-doping, unique microstructure, modified conductive agent super-p, and new types of electrolytes for low-temperature electrochemical performance^9,15–19^. References^9,15–19^ indicated that the appropriate particle size, large specific surface area, fewer contact points between particles, and high electrode conductivity could effectively enhance the low-temperature electrochemical performance to a certain extent. However, the mechanisms for the electrochemical performance of the LTO electrode decreasing at low temperatures were rarely explored. Thus, in the current study, the low-temperature electrochemical performance of LTO and LTO–RTO anode materials was investigated, and its mechanisms were explored by analyzing the influence of electrolytes, crystal structure variation of the anode material, lithium ion diffusion path, lithium ion diffusion efficiency, and activation energy, among others. Moreover, the improved methods were presented by reducing the melting point of the liquid electrolyte or by coating an RTO layer on the surface of the LTO nanosheet.

## Results and Discussion

### Characteristics of the crystal structure of as-prepared anode materials

**Figure 1:**
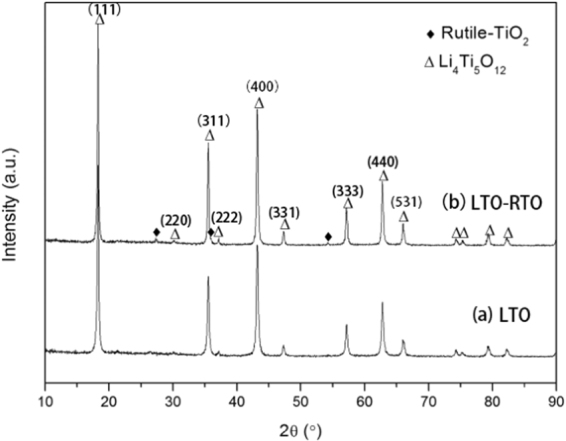
XRD patterns of as-prepared LTO powder (**a**) and LTO–RTO powder (**b**).

Figure 1 shows the XRD patterns of the LTO powder and the LTO–RTO powder. The patterns suggest that all diffraction peaks of LTO are in accordance with a cubic spinel structure with the Fd-3 m space group (JCPDS: 49–0207); however, some weak diffraction peaks in the pattern of LTO–RTO were detected at 27.5°, 36.1°, 54.3°, which correspond to rutile-TiO_2_ (JCPDS: 21–1276). These findings indicate that the composite consisted of Li_4_Ti_5_O_12_ and rutile-TiO_2_.

**Figure 2:**
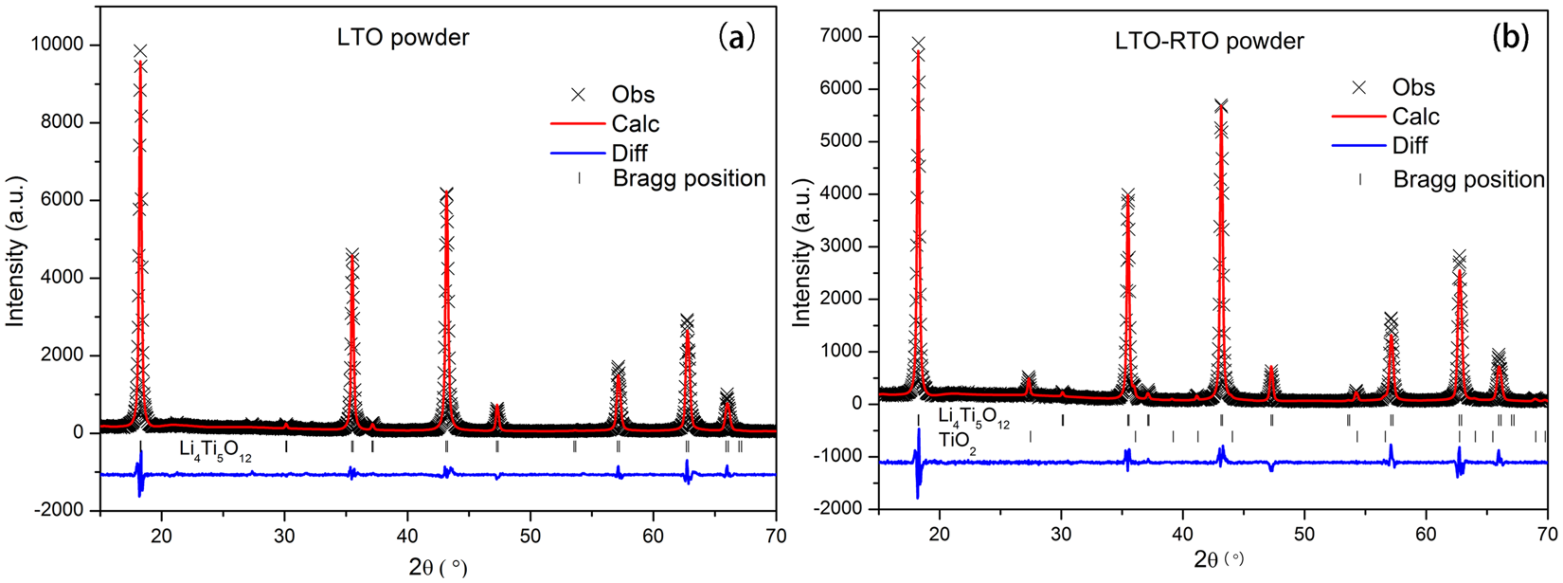
Rietveld refinement patterns of the LTO powder (**a**) and the LTO–RTO powder (**b**).

To elucidate the crystalline structures of LTO and LTO–RTO, as well as ascertain the rutile-TiO_2_ content in the LTO–RTO crystals, Rietveld refinement was conducted. Profile (R_p_), weighted profile residuals (R_wp_), and goodness of fit (χ^2^) are used to estimate refinement results. The result is generally reasonable when R_p_, R_wp_, and χ^2^ are less than 6%, 10%, and 3. The results of Rietveld refinement are presented in Fig. 2 and Table 1 based on a model of Li_1_ atoms occupying the 8a (1/8, 1/8, 1/8) position, Li_2_/Ti atoms occupying the 16d (1/2, 1/2, 1/2) position, and O atoms occupying the 32e (*x*, *x*, *x*). The results showed that the RTO content in the LTO–RTO powder was 3.257 wt%. Several significant differences in structure were observed between LTO and LTO–RTO. Compared with LTO–RTO, LTO had larger lattice parameter (a), lattice volume (V), oxygen coordinate (*x*), Li_1_-O bond distance, and Li_1_-Li_1_ bond distance but smaller Li_2_/Ti-O bond distance. These differences indicated the presence of larger tetrahedrons and smaller octahedrons in LTO and the opposite in LTO–RTO^20^. The number of free octahedral voids determined the Li-insertion capacity at the potential range of 1–2.5 V^21^, and the smaller value of *x* suggested decreasing structural distortion^22^. Thus, LTO–RTO electrodes are predicted to exhibit a better electrochemical performance compared with the LTO electrode when Li ion diffusion is from 8a-16c along the <011> direction.

**Table 1:** Structural parameters of the LTO and LTO–RTO crystals.

Sample	a, nm	V, nm^3^	*x*	Li_1_-O, nm	Li_2_/Ti-O, nm	Li_1_-Li_1_	wt (RTO)%	Rp, Rwp, χ^2^
LTO	8.361737(2)	584.641	0.261786	1.98107(2)	1.99675(2)	3.621	/	6.47%, 8.33%, 2.449
LTO–RTO	8.356956(2)	583.639	0.258768	1.93625(2)	2.01863(3)	3.619	3.257	8.39%, 10.63%, 2.724

### Low-temperature electrochemical performance

**Figure 3:**
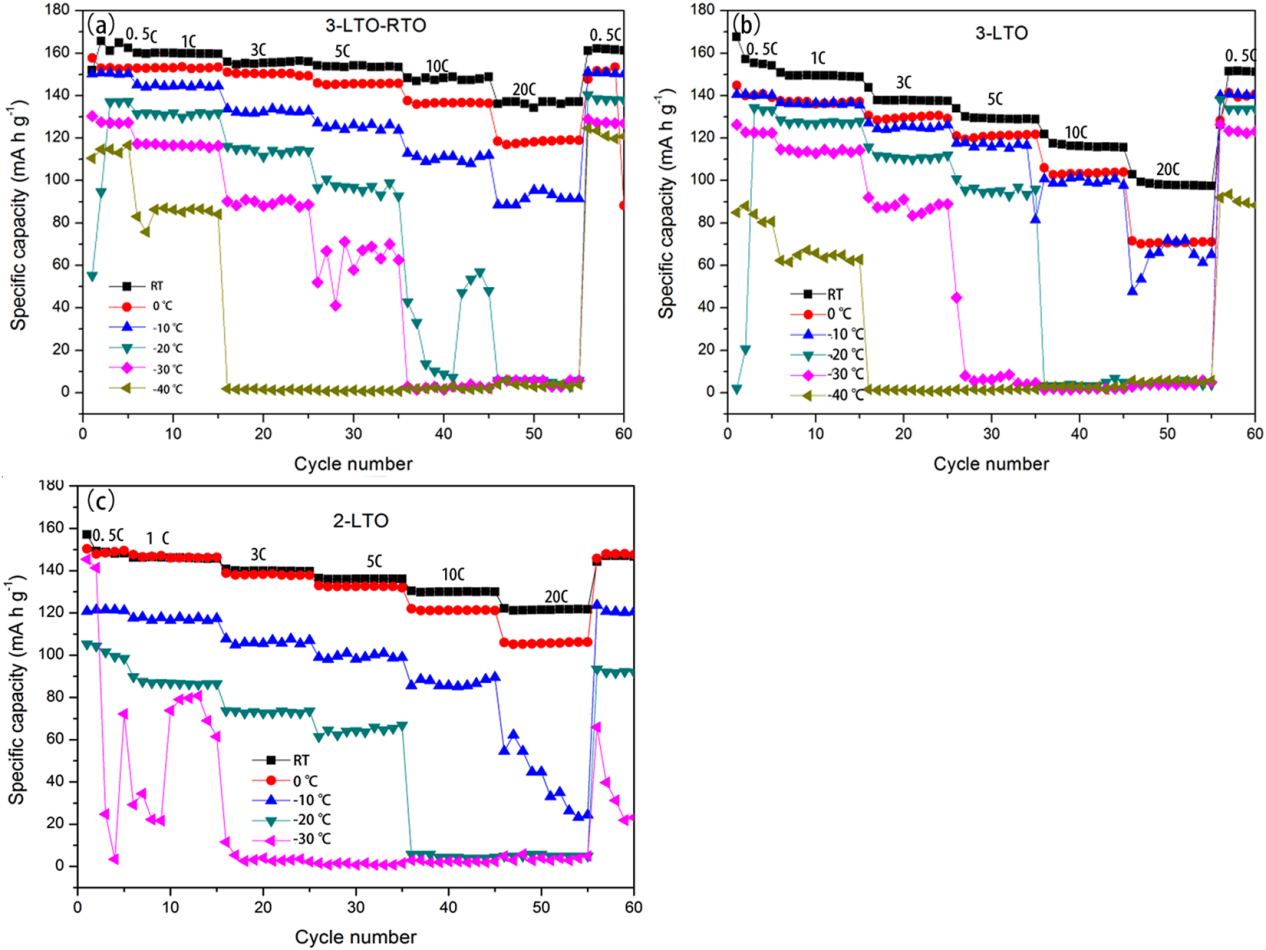
The electrochemical performances of (**a**) 3-LTO-RTO, (**b**) 3-LTO, and (**c**) 2-LTO at different temperatures.

Figure 3 shows the electrochemical performance of the LTO and LTO–RTO electrodes between room temperature (RT) and −40 °C. The LTO electrode and the LTO–RTO electrode cycled in 1 M LiPF_6_ ethylene carbonate (EC) + ethyl methyl carbonate (EMC) + dimethyl carbonate (DMC) ternary electrolyte (volume ratio, 1:1:1) are identified as 3-LTO, 3-LTO-RTO, respectively. Similarly, the LTO electrode cycled in 1 M LiPF_6_ EC + DMC binary electrolyte (volume ratio, 1:1) is identified as 2-LTO. The electrochemical performance of cells lowered with a decrease in temperature regardless of the electrode materials and the electrolytes of cells. Compared with the electrochemical performance of 3-LTO-RTO and 3-LTO at different temperatures, the LTO–RTO electrode exhibits the better electrochemical performance at any testing temperature. Under room-temperature conditions, the discharge specific capacity of the LTO electrode are 155 and 165 mAh g^−1^ at 0.5 C, respectively, whereas those of the LTO–RTO electrode are 149 and 162 mAh g^−1^ at 1 C, respectively. At −40 °C, the discharge specific capacity of the LTO electrode are 86 and 65 mAh g^−1^ at 0.5 and 1 C, respectively, whereas those of the LTO–RTO electrode are 115 and 86 mAh g^−1^ at 0.5 and 1 C, respectively. At −40 °C, 55.48% and 43.62% of the discharge capacity of the LTO electrodes are maintained at 0.5 C, whereas 69.70% and 51.58% of the discharge capacity of the LTO–RTO electrodes are maintained at 1 C. At −40 °C, the superior low-temperature capacity of the LTO–RTO electrode should be related to the intrinsic characteristics of the electrode material.

Mechanisms on the reduction of the electrochemical performances of the LTO–based electrodes at low temperature and the reasons of the LTO–RTO electrodes exhibit higher specific capacities compared with the LTO electrodes are explored.

### Effect of electrolyte

The electrochemical performances of the LTO and LTO–RTO electrodes in the ternary electrolyte apparently declined when cycled at −40 °C. The LTO electrodes in the binary electrolyte almost failed to charge and discharge below −30 °C. Meanwhile, the electrochemical performance of the LTO–based electrodes in the ternary electrolyte was superior to that of the LTO electrodes in the binary electrolyte below 0 °C. Data on the electrochemical performance of LTO in different electrolytes are presented in Figure S1. These results indicate that the sharp decrease in low-temperature electrochemical performance of LTO–based electrodes is most likely related to the electrolyte. And the ternary electrolyte can be more suitably used at low temperatures. The viscosity and ionic conductivity of the liquid electrolytes are widely regarded as key factors influencing the electrochemical performance of batteries^23,24^. However, viscosity increases, whereas ionic conductivity decreases when the electrodes are cycled at low temperatures^24^. The melting points of EC, DMC, and EMC are 37 °C, 3 °C, and −55 °C, respectively. The proportion of EC in the binary electrolyte is 50 vol.%, which is greater than that in the ternary electrolyte (about 33 vol.%). Thus, the electrolyte using EC and DMC as the solvent is easier to crystallize and exhibits higher viscosity and lower ionic conductivity, compared with the electrolyte using EC, DMC, and EMC as the solvent at low temperatures. The freezing point of the ternary electrolyte is ~−50 °C and that of the binary electrolyte is ~−30 °C. The conductivity of the ternary electrolyte is 4~10 × 10^−3^ S cm^−1^ and ~2 × 10^−3^ S cm^−1^ at 20 °C and −20 °C, respectively. And the conductivity of the binary electrolyte is 8.8 × 10^−3^ S cm^−1^ and 0.58 × 10^−3^ S cm^−1^ at 20 °C and −20 °C, respectively^25,26^. Thus, lithium ion diffusion in the electrolyte and charge transfer between the electrode/electrolyte interfaces become more difficult. This analysis indicates that the good electrochemical performance in LTO–based materials in ternary electrolyte is attributed to lower viscosity and higher ionic conductivity at low temperatures. Therefore, reducing the melting point of liquid electrolytes can potentially improve performance at low temperatures. On the basis of the results of the electrolyte, only the ternary electrolyte was adapted to proceed with the subsequent investigations.

### Variation in crystal structure during cycling at low temperatures

**Figure 4:**
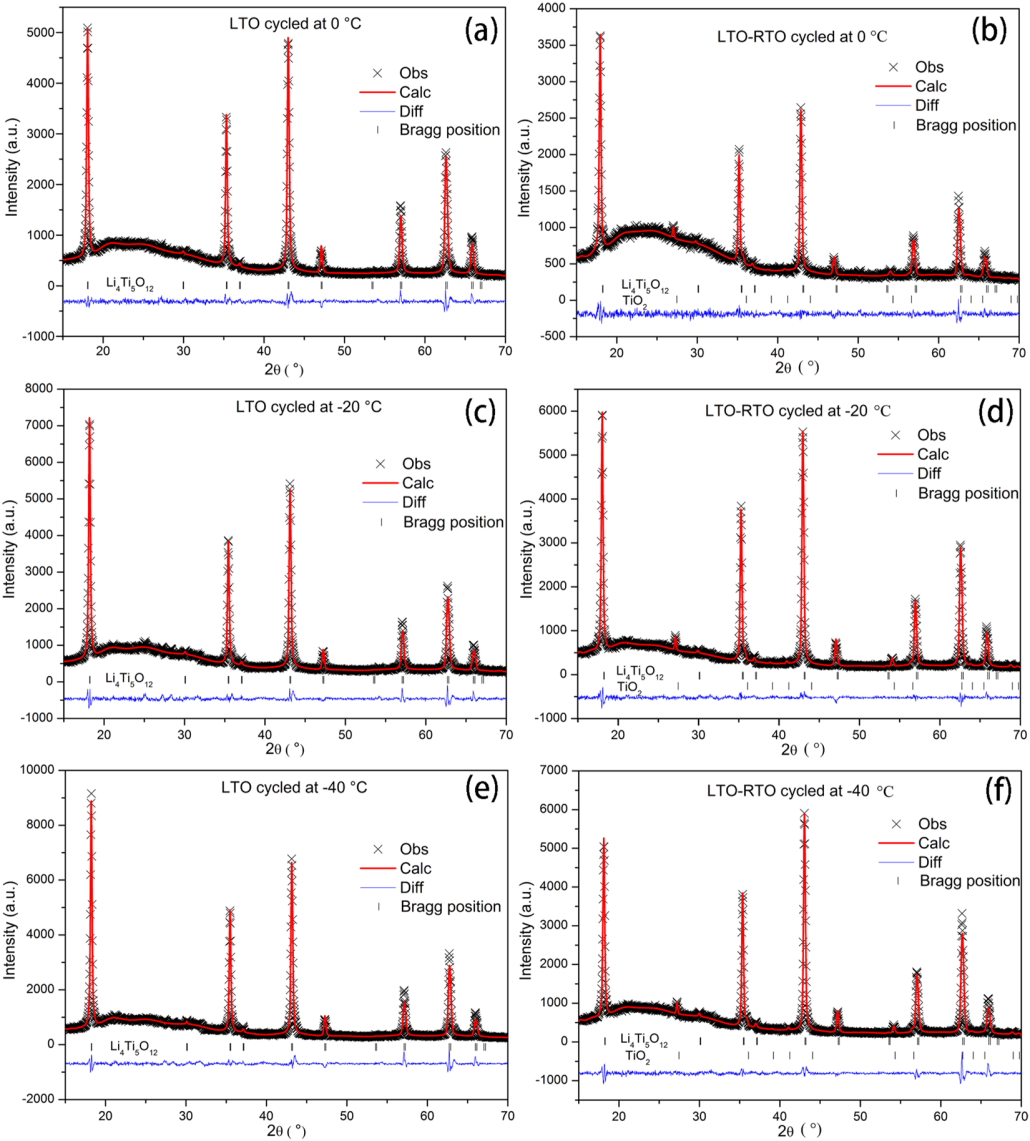
Rietveld refinement patterns of the cycled LTO powder at 0 °C (**a**), −20 °C (**c**), and −40 °C (**e**) as well as the cycled LTO–RTO powder at 0 °C, (**b**), −20 °C, (**d**), and −40 °C (**f**).

Figure 4 shows the Rietveld refinement patterns of the cycled LTO and LTO–RTO electrodes after charge and discharge at 0 °C, −20 °C, and −40 °C, respectively. The values of R_wp_, R_p_, and χ^2^ for all samples are almost less than 10%, 6%, and 3, respectively, which verifies the reliability of the Rietveld refinement results. The structural parameters obtained from Rietveld refinement are listed in Table 2.

**Table 2:** Structural parameters of LTO powder and LTO–RTO powder cycled 15 times at 0 °C, −20 °C, and −40 °C.

Sample	a, nm	V, nm^3^	*x*	Li_1_-O, nm	Li_2_/Ti-O, nm	Li_1_-Li_1_, nm	Rp, Rwp, χ^2^
LTO~0 °C	8.350098	582.203	0.258519	1.93106(2)	2.01890(3)	3.616	4.58%, 6.09%, 1.752
LTO~−20 °C	8.352905	582.791	0.259828	1.95064(2)	2.00949(3)	3.617	4.98%, 6.50%, 2.281
LTO~−40 °C	8.351963	582.594	0.258546	1.93188(2)	2.01914(3)	3.617	5.53%, 7.61%, 3.052
LTO–RTO~0 °C	8.346919	581.539	0.260629	1.96083(3)	2.00194(4)	3.614	3.62%, 4.66%, 1.281
LTO–RTO~−20 °C	8.349110	581.997	0.258189	1.92606(1)	2.02122(2)	3.615	3.82%, 4.60&, 1.524
LTO–RTO~−40 °C	8.349153	582.006	0.260355	1.95739(2)	2.00457(3)	3.615	5.36%, 7.26%, 2.131

All parameters of the crystal structure change irregularly, regardless of whether such parameters refer to the LTO or LTO–RTO; However, the parameters of the lattice and the Li_1_-Li_1_ bond length related to lithium ion diffusion decrease, compared with those of the un–cycled powder (Table 1). The studies by Laumann *et al*. and Dolotko *et al*.^20,27^ indicate that the changes in the crystal structure of Li_4_Ti_5_O_12_ without charge/discharge are regular whether at high or low temperatures. Consequently, the structure is affected not only by temperature but also by lithium ion insertion/extraction during charge/discharge. Dolotko *et al*.^20^ suggested that the lithium ion diffusion path is more probable along 8a-32e-32e-8a rather than 8a-16c-8a by valence bond theory at low temperatures. Therefore, it is speculated that the lithium ion diffusion path of Li_4_Ti_5_O_12_–based materials could change at low temperatures. The diffusion path of lithium ions could significantly affect low-temperature electrochemical performance, but this has yet to be verified by further experimentation. Ideally, lithium ion intercalated/deintercalated processes are fully reversible, and the structure returns to the original state after charging. However, the truth deviates from the ideal, particularly charging/discharging under the condition of high rates or low temperatures. Lithium ions cannot fully intercalate while discharging nor deintercalate while charging, Consequently, lithium ions accumulate continually in octahedral 16c positions and tetrahedral 8a positions (Lithium ions in Li_4_Ti_5_O_12_ are believed to be along the [011] orientation diffusing from 8a-16c-8a during the charge/discharge process^28,29^.). The XRD peak intensities of (311) and (400) are related to the number of lithium ions at 8a (tetrahedron) and 16c (octahedron) sites, respectively^21^. Therefore, a simple method can be used to investigate the diffusion path of lithium ions. The XRD peak intensity ratios of (311)/(111) and (400)/(111) (referred to as I_(311)_/I_(111)_ and I_(400)_/I_(111)_, respectively) gained from the measured XRD patterns (Fig. 4) are listed in Table 3. For the cycled LTO–RTO electrode, the values of I_(311)_/I_(111)_ and I_(400)_/I _(111)_ increased when the tested temperatures decreased from 0 °C to −40 °C. However, for the cycled LTO electrode, the opposite result was obtained. The former conformed to the regulations, which lithium ions accumulated continually in octahedral 16c and tetrahedral 8a positions, and the diffusion path remained unchanged along with 8a-16c-8a. However, the latter indicated that the diffusion path of lithium ions in the LTO electrode had been changed. Combined with the results obtained by Dolotko *et al*.^20^, the diffusion path of lithium ions may be along 8a-32e-32e-8a. Given the low-temperature electrochemical performance of the LTO and LTO–RTO electrodes (Fig. 3), the conclusion is that diffusion of lithium ions is more difficult and requires more energy in the LTO electrode than in the LTO–RTO electrode. Therefore, on the basis of the crystal structure, the change in diffusion path in the LTO electrodes resulting from the change in structural parameters during cycling at low temperatures could reduce the electrochemical performance of the LTO electrodes. By contrast, for the LTO–RTO electrode, the main diffusion path of lithium ions remains along 8a-16c-8a, according to results listed in Table 3. Thus, the variation in crystal structure is not sufficient to contribute to the reduction in electrochemical performance at low temperatures.

**Table 3:** Intensity ratios of (400)/(111) and (311)/I(111) for the cycled LTO and LTO–RTO electrodes at 0 °C, −20 °C, and −40 °C.

Sample	I_(311)_/I_(111)_	I_(400)_/I_(111)_
LTO~0 °C	0.67	1.112
LTO~−20 °C	0.55	0.882
LTO~−40 °C	0.57	0.860
LTO-RTO~0 °C	0.52	0.839
LTO-RTO~−20 °C	0.68	1.111
LTO-RTO~−40 °C	0.83	1.388

### Dynamical features of LTO–based anode materials cycled at low temperatures

**Figure 5:**
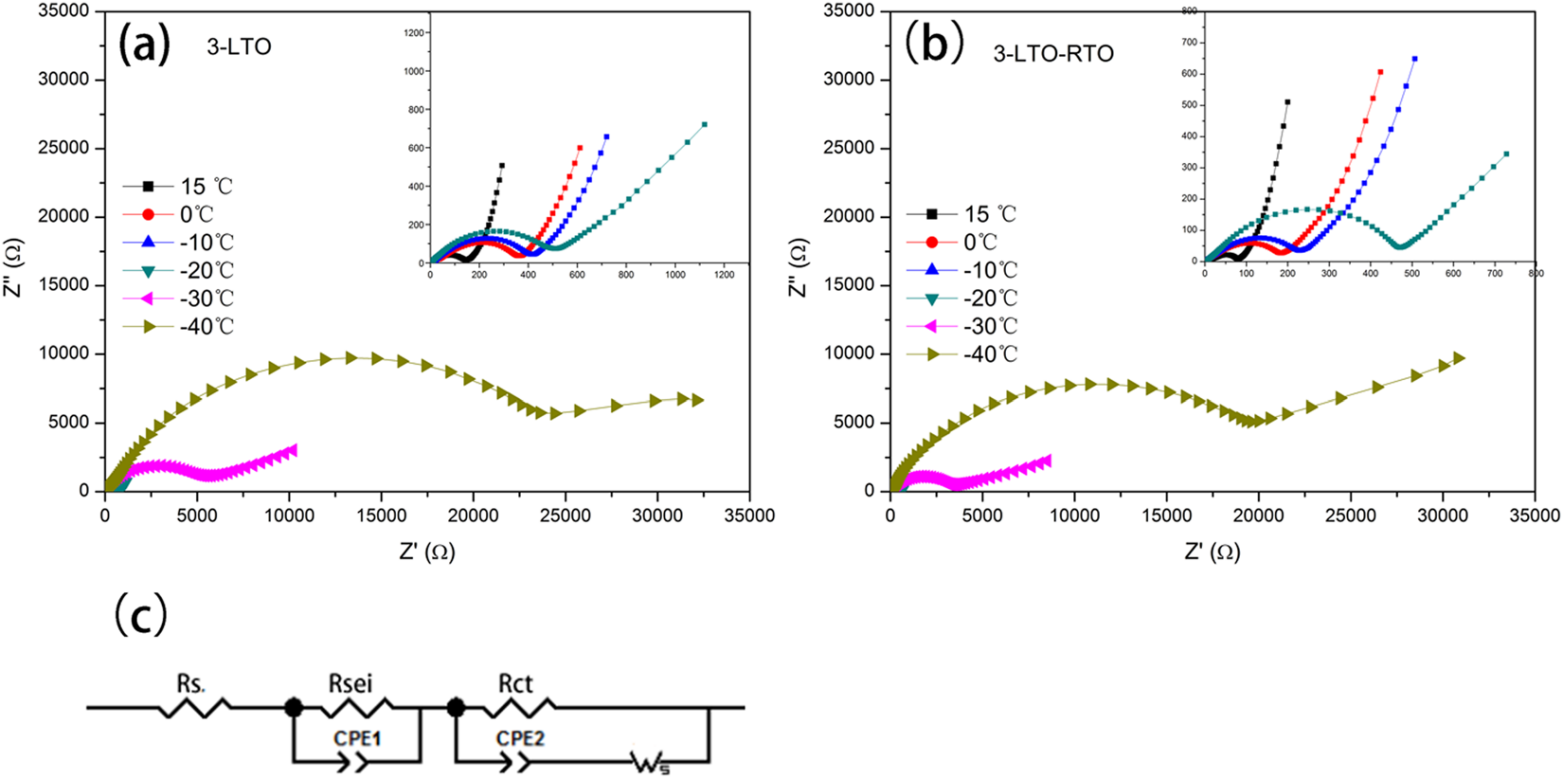
Electrochemical impedance spectroscopy (EIS) of 3-LTO composite (**a**), 3-LTO-RTO (**b**) and the equivalent circuit diagram (**c**).

The electrochemical impedance spectra (EIS) of LTO–RTO composites and LTO electrodes cycled 3 times at 0.5 C was measured at different temperatures. The results are presented in Fig. 5. As shown, all Nyquist plots consist of the real-axis intercept at a high frequency, a minimal depressed semicircle and a large depressed semicircle from a high-frequency range to a medium-frequency range, and a slope line about 45° at a low-frequency range, which corresponded to the ohmic resistance (R_s_), charge transfer resistance (R_ct_), and the Warburg impedance (W_s_), successively, except for the first depressed semicircle. The first depressed semicircle is usually associated with the resistance of the solid electrolyte interface (SEI) film (R_sei_)^30^. SEI film was previously thought to be impossible to generate in the potential range of 1–2.5 V in Li_4_Ti_5_O_12_-based anode materials. However, the SEI film has been recently proved in Li_4_Ti_5_O_12_-based electrodes in the potential range of 1–2.5 V^31,32^. To further ascertain the existence of the SEI film under this condition, the SEM and FTIR spectra of 3-LTO before and after cycling were obtained. The results are presented in Figure S2. SEI film clearly exists on the electrode surface, as determined from the SEM images. In Figure S2(d), characteristic peaks of Li_2_CO_3_ (1488, 1434, and 865 cm^−1^) and ROCO_2_Li (1627 cm^−1^) are found in all electrodes. This finding presents a direct evidence for the existence of the SEI film. Moreover, the intensity of these characteristic peaks decreased after cycling. Li_2_CO_3_ and ROCO_2_Li generated in the electrode without cycling may be attributable to the existence of trace amounts of HF and H_2_O in the electrolyte, which mainly causes the formation of an SEI film^31^. As the cycle proceeds, the SEI film may be dissolved and then formed repeatedly under the action of Ti^3+^ catalysis, which can increase the SEI film resistance^31^. This condition may be more serious with a decrease in temperature.

On the basis of the aforementioned analysis, the corresponding equivalent circuit diagram is chosen and shown in Fig. 5(c). The impedance parameters are listed in Table 4. With a reduction in the temperature, R_s_, R_sei_ and R_ct_ increase gradually. R_s_ is mainly related to electrolyte resistance, whereas R_sei_ reflects film resistance. An increase in R_sei_ indicates that a side reaction is aggravated. The value for R_sei_ of the LTO electrodes is larger than that of the LTO–RTO electrodes at the same testing temperature, suggesting that the side reaction of LTO–RTO electrodes was suppressed to a certain extent. Above three resistances, the value of R_ct_ is far greater than those of the two others. The charge transfer resistance between the electrode and the electrolyte is weighted through the value of R_ct_. The increase in R_ct_ indicates that the interface dynamic activity of cells decreases when cycling occurs at low temperatures.

**Table 4:** Impedance parameters and D_Li_ of 3-LTO-RTO and 3-LTO.

Sample	Temperature (°C)	R_s_ (Ω)	R_sei_ (Ω)	R_ct_ (Ω)	R (Ω)	D_Li_ (cm^2^ s^−1^)
3-LTO-RTO	15	2.7	6.7	67.7	77.1	1.96 × 10^−14^
	0	4.7	25.7	181.9	212.3	4.46 × 10^−15^
	−10	5.1	50.7	234.1	289.9	3.10 × 10^−15^
	−20	5.2	65.0	455.0	525.2	1.65 × 10^−15^
	−30	16.9	254.1	3779.0	4050.0	2.01 × 10^−17^
	−40	20.1	463.8	20600.0	21083.9	2.81 × 10^−18^
3-LTO	15	5.2	7.8	142.2	155.2	1.49 × 10^−14^
	0	5.5	26.7	353.0	385.2	3.11 × 10^−15^
	−10	6.5	58.3	401.0	465.8.	1.98 × 10^−15^
	−20	7.7	136.4	513.5	657.6	3.66 × 10^−16^
	−30	21.6	394.3	5758.0	6173.9	4.54 × 10^−18^
	−40	23.5	514.7	268300.0	27368.2	3.57 × 10^−19^

Meanwhile, the bulk dynamics activity is weighted through the efficiency of lithium ion diffusion (D_Li_) at different temperatures. The calculated process of D_Li_ and the Z′~ω^−1/2^ plots (Figure S3) are presented in Supplementary Information. The results of the diffusion coefficient of the LTO and LTO–RTO electrodes are listed in Table 4. As shown, the lithium ion diffusion coefficients of LTO–based anode materials decreased with a decrease in temperature, indicating that the bulk dynamic activity of active materials decreases at low temperatures. Meanwhile, the D_Li_ value of LTO–RTO active materials is greater than that of LTO active materials at the same temperature. Therefore, LTO–RTO presents an advantage over the LTO in this aspect.

To confirm the advantage of the LTO–RTO electrode in terms of lithium ion diffusion, activation energy was calculated based on the lithium ion diffusion coefficient. The detailed calculation and the plotting of D_Li_~1000/T (Figure S4) are presented in Supplementary Information. The calculated activation energy of 3-LTO-RTO was 41.64 KJ/mol and that for 3-LTO was 60.99 KJ/mol. The activation energy of 3-LTO-RTO was markedly lower than that of 3-LTO at low temperatures, which proved that lithium ion diffusion was easier in the 3-LTO-RTO electrode than in the 3-LTO electrode. This finding confirms the previous deduction that the lithium ion diffusion path in the LTO–RTO electrode remained unchanged along the 8a-16c-8a path; however, the lithium ion diffusion path in the LTO electrode can change, and lithium ions can be diffused toward a difficult path where a greater activation energy is required.

**Figure 6:**
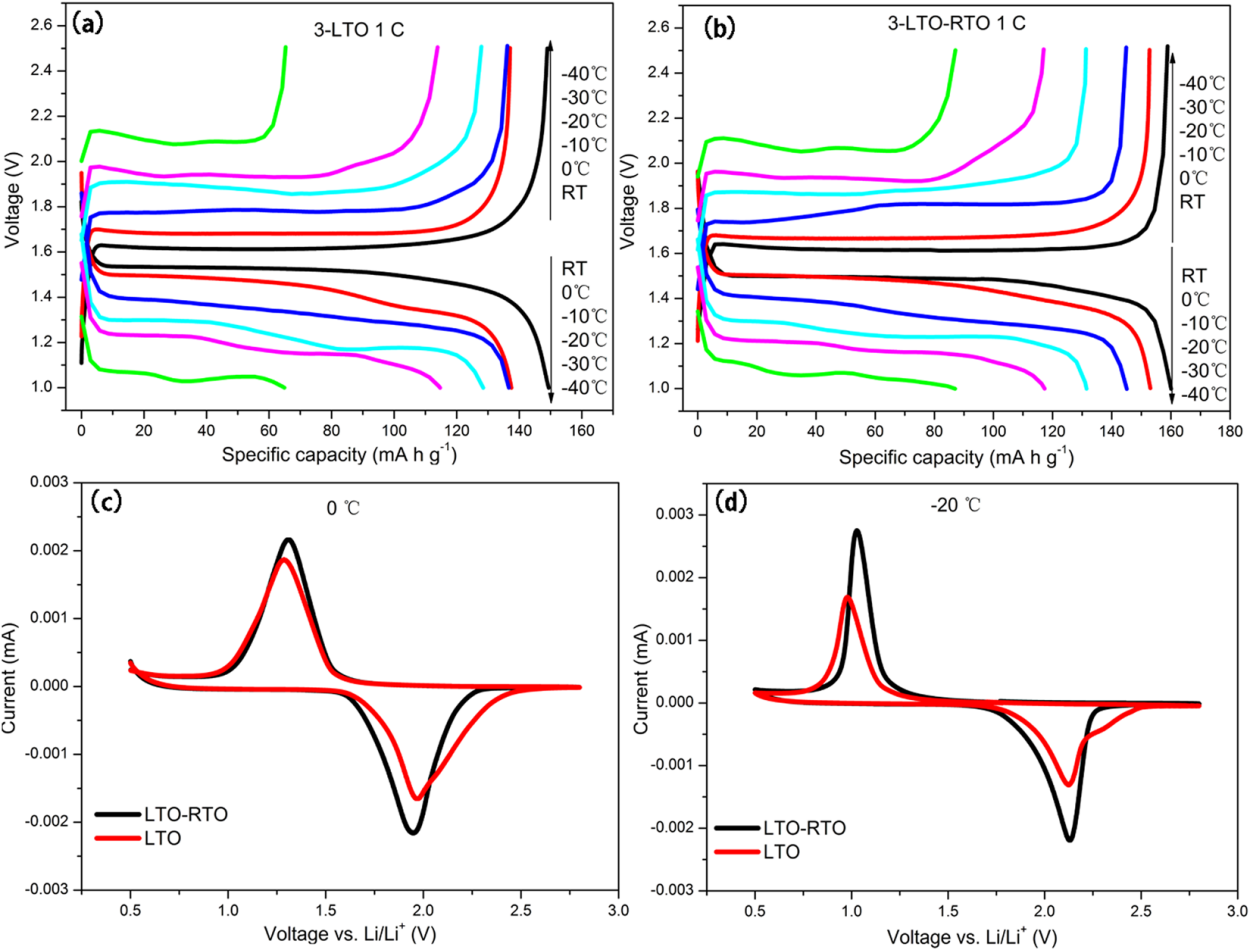
Charge/discharge curves of the LTO (**a**) and LTO–RTO (**b**) electrodes; CV curves of the LTO–RTO and LTO electrodes at 0 °C (**c**) and −20 °C (**d**), respectively.

To further investigate the dynamics of lithium ion diffusion in the bulk, the voltage difference between the charge platform and the discharge platform (ΔE) and the voltage difference between the oxidation peak and the reduction peak (ΔV) were measured^33,34^. Figure 6(a) and (b) show the charge/discharge voltage curves of the LTO and LTO–RTO electrodes at 1 C from room temperature to −40 °C. Figure 6(c) and (d) present the CV curves of the LTO and LTO–RTO electrodes at 0 °C and −20 °C. The ΔE value of both LTO–RTO and LTO increased gradually with a decrease in temperature, indicating that the polarization increased while the dynamics of lithium ion diffusion decreased with a decrease in temperature. The ΔV value of the LTO–RTO electrode was smaller than that of the LTO electrode, suggesting that the LTO–RTO electrodes had better lithium ion diffusion dynamics than the LTO electrodes.

Briefly, by the analyses of R_sei_, R_ct_, D_Li_, activation energy, ΔE, and ΔV from the perspective of the dynamics, these results indicate that with a decrease in temperature, a side reaction is aggravated; in addition, the rate of charge transfer between electrolytes and electrodes and the lithium ion diffusion coefficient in the bulk decrease when LTO–based anode materials are cycled at low temperatures. These occurrences lead to the degradation of electrochemical performance (Fig. 3). However, the lithium ion diffusion coefficient of LTO–RTO active materials is greater than that of LTO active materials at the same temperature; the LTO–RTO electrodes have better lithium ion diffusion dynamics than the LTO electrodes; and the activation energy of the LTO–RTO electrodes on the basis of the lithium ion diffusion coefficient is less than that of the LTO electrodes. All of these findings help explain why the LTO–RTO electrode exhibits better electrochemical performance than the LTO electrode.

## Conclusion

On the basis of the aforementioned findings, the following results are obtained.

With a decrease in temperature, the electrochemical performance of Li_4_Ti_5_O_12_–based anode materials decreased at low temperatures (e.g., 0 °C–−40 °C). Compared with that at room temperature, the capacity retention rates of the LTO electrode at −40 °C were 55.48% at 0.5 C and 43.62% at 1 C. In addition, the LTO–RTO electrode exhibited capacity retention rates of 69.70% and 51.58% under the same condition. The LTO–RTO electrode exhibited a better electrochemical performance than the LTO electrode.

First, the decrease in the low-temperature electrochemical performance of the Li_4_Ti_5_O_12_–based electrodes is influenced by the electrolyte. The viscosity of the electrolyte increases and the ionic conductivity of the electrolyte is reduced when the cell is cycled at low temperatures. Moreover, the electrolyte using a high proportion of EC or DMC as the solvent is easier to crystallize at low temperatures because of the higher melting points of EC (37 °C) and DMC (3 °C). Consequently, the electrochemical performance of the Li_4_Ti_5_O_12_-based electrodes at low temperatures decreases.

Second, the decrease in the low-temperature electrochemical performance of the Li_4_Ti_5_O_12_–based electrodes is related to changes in the crystal structure. When cycling at low temperatures, the crystal structure parameters the of Li_4_Ti_5_O_12_-based electrodes change; the lattice parameters and the Li_1_-Li_1_ bond length related to lithium ion diffusion decrease at low temperatures. On the basis of these changes in the crystal structure, for the LTO electrode, the lithium ion diffusion path of 8a-16c-8a changes. The 8a-32e-32e-8a path might have hindered the lithium ion diffusion. However, variations in the crystal structure parameters of the LTO–RTO electrode failed to sufficiently change the lithium ion diffusion path. Thus, the LTO–RTO electrode exhibited a better electrochemical performance compared with the LTO electrode when cycling was performed at low temperatures.

Finally, from the perspective of dynamics, the results for R_sei_, R_ct_, D_Li_, ΔE, and ΔV indicate that the dynamics of the Li_4_Ti_5_O_12_ anode materials decrease with a decrease in temperature, thereby reducing the electrochemical performance of the Li_4_Ti_5_O_12_–based electrode at low temperatures. However, the active energy based on lithium ion diffusion of the LTO–RTO electrode was lower than that of the LTO electrode. This result also explains why the LTO–RTO electrode exhibited a better electrochemical performance at low temperatures, compared with the LTO electrode.

## Methods

### Material synthesis

Spinel LTO and LTO–RTO were synthesized using the gel–hydrothermal method with lithium hydroxide monohydrate (LiOH•H_2_O) as the lithium source and titanium (IV) butoxide (Ti(C_4_H_9_O)_4_) as the titanium source. Firstly, 16.25 g of Ti(C_4_H_9_O)_4_ was dissolved in 34 mL alcohol as A. Secondly, 1.68 g of LiOH•H_2_O was dissolved in 20 mL of distilled water as B. Then, solution B was dropped slowly into solution A with magnetic stirring. The gained white suspension was transferred into a 100 mL stainless-steel autoclave and then reacted at 180 °C for 36 h. After filtrating and drying at 80 °C for 7 h, precipitate was calcined at 600 °C for 10 h and transferred into final product. By changing the amount of Ti(C_4_H_9_O)_4_ to 15.10 g, the pure LTO was obtained using the same procedure.

### Electrode fabrication and coin cell assembly and disassembly

LTO or LTO-RTO compound, acetylene black, polyvinylidene (weight ratio, 80:10:10) were dissolved in n-methyl pyrrolidinone to form a slurry, coated on a copper foil substrate, and dried at 80 °C for 12 h. The prepared copper foil was cut into small discs with an area of 1.58 cm^2^. CR2025 coin cells were assembled in an Ar-filled glove box (H_2_O < 0.1 ppm and O_2_ < 4 ppm) by using prepared discs as the positive electrode, metallic lithium as the counter electrode, a microporous polyethylene film as the separator, and 1 M LiPF_6_ EC + EMC + DMC as the ternary electrolyte (the volume ratio, 1:1:1) or 1 M LiPF_6_ EC + EMC as the binary electrolyte (volume ratio, 1:1) as the electrolyte. The cells were disassembled and then washed in the DMC several times.

### Structural characterization

The crystalline phases of the samples were characterized by X-ray diffraction (Rigaku DMAX2000) with Cu k_α_ radiation. Data for crystalline phase identification were collected by continuous scanning at 2θ = 10°–90°. Data for phase structure refinement were collected by step scanning at 2θ = 15°–70° with a step of 0.03° and a counting time of 2 s per step. Rietveld refinements were performed using the GSAS program with the EXPGUI interface. During the entire process, background parameters, zero, polar, lattice parameters, atomic fractional coordinates, atomic isotropic displacement parameters, phase fractions, profile parameters, and SH Pref Orient parameters were fitted using the shifted Chebyshev function as the background function and the Pearson VII function as the profile function.

### Electrochemical characterization

The galvanostatic charge and discharge test, with a potential range of 1–2.5 V (vs. Li/Li^+^), was executed by LAND CT2001A from room temperature to −40 °C. Before testing the electrochemical performance, all cells were placed on their corresponding testing temperatures for 4 h. Data were obtained by electrochemical impedance spectroscopy (EIS) using an electrochemical workstation (CHI660E) at 10^−2^–10^5^ Hz frequency range. Data were collected by cyclic voltammetry (CV) with a scan rate of 5 mV s^−1^ within the voltage range of 0.5–2.5 V by using an electrochemical workstation (CHI600E).

### Morphological and constituent characterization of SEI film

Surface morphology of the LTO electrodes cycled in the ternary electrolyte was characterized by field emission scanning electron microscopy (SEM, Hitachi S-4800). The surface group constituent of the cycled LTO electrodes was characterized in the ternary electrolyte by Fourier transform infrared spectroscopy (FTIR, TENSOR 27) between 200–2000 cm^−1^.

## Electronic supplementary material

Mechanisms of the decrease in low-temperature electrochemical performance of Li4Ti5O12-based anode materials
