# Supplementary material for: Mechanisms of the decrease in low-temperature electrochemical performance of Li4Ti5O12-based anode materials
Source: Sci Rep. 2017 Nov 10;7:15292. doi: 10.1038/s41598-017-15504-4 (PMC5681549; doi:10.1038/s41598-017-15504-4)
Supplement: Supplementary file 1 — Mechanisms of the decrease in low-temperature electrochemical performance of Li4Ti5O12-based anode materials [file 41598_2017_15504_MOESM1_ESM.docx]

Mechanisms of the decrease in low-temperature electrochemical performance of Li_4_Ti_5_O_12_-based anode materials

Qian Huang^1^, Zhen Yang^1^, Jian Mao^1,*^

*^1^Sichuan University, College of Materials Science and Engineering, Chengdu, 610064, China*

*^*^maojian@scu.edu.cn*

^
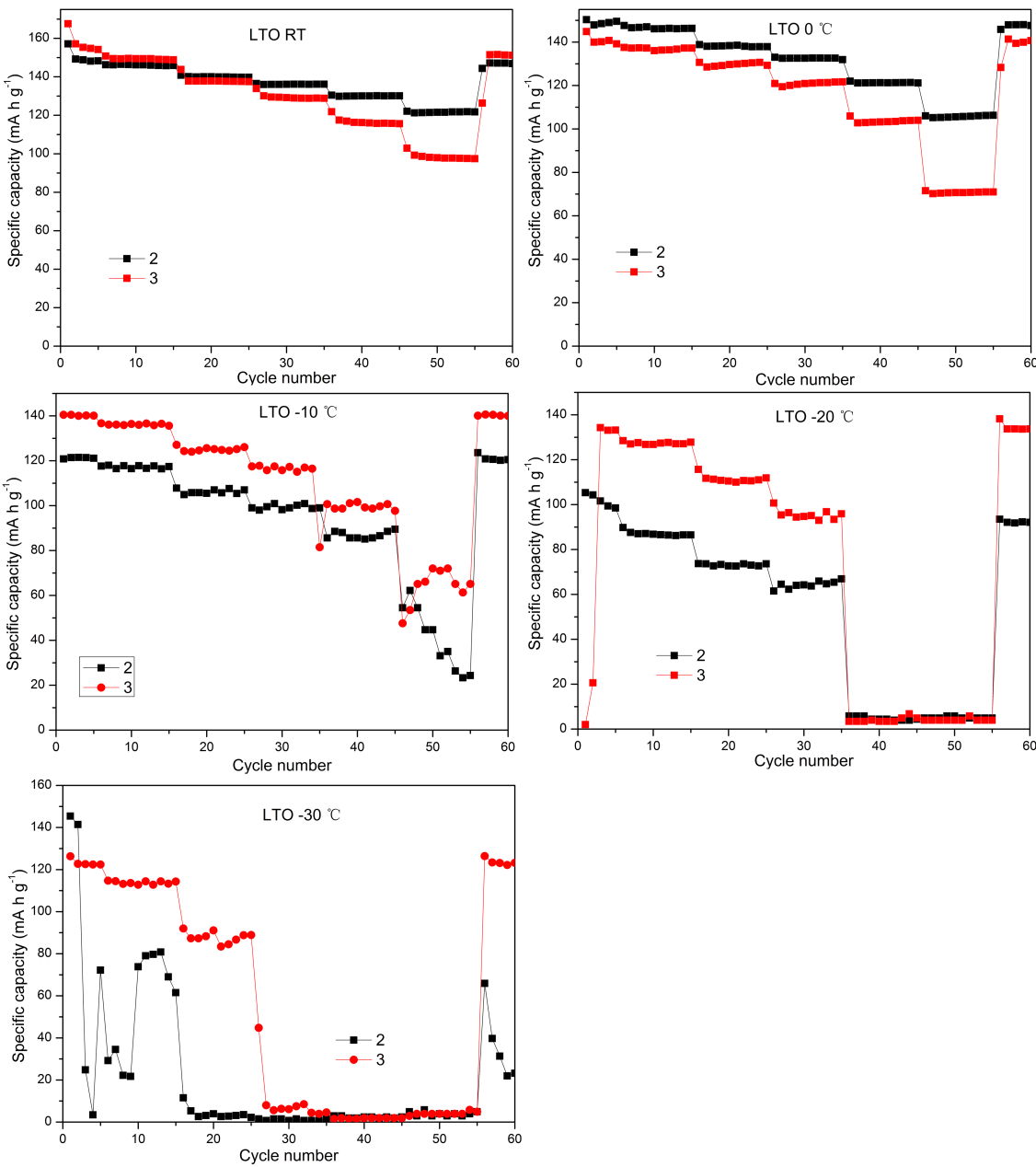
^

**Figure S1** The electrochemical performance of LTO in different electrolytes and at different temperatures.


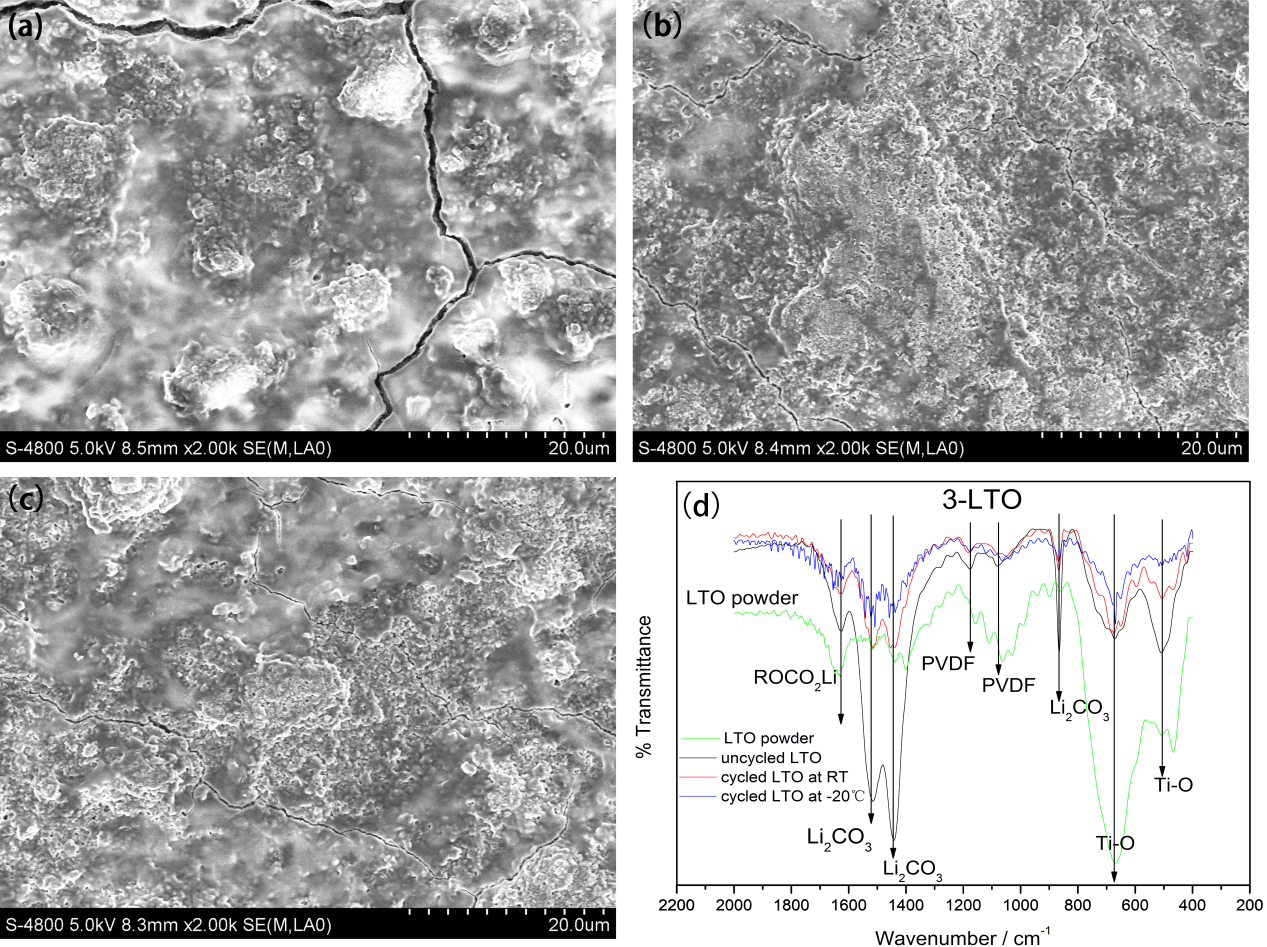


**Figure S2** SEM images of uncycled LTO (a), LTO cycled at RT (b) and cycled at -20 °C (c), and FTIR spectra of the LTO powder (d).


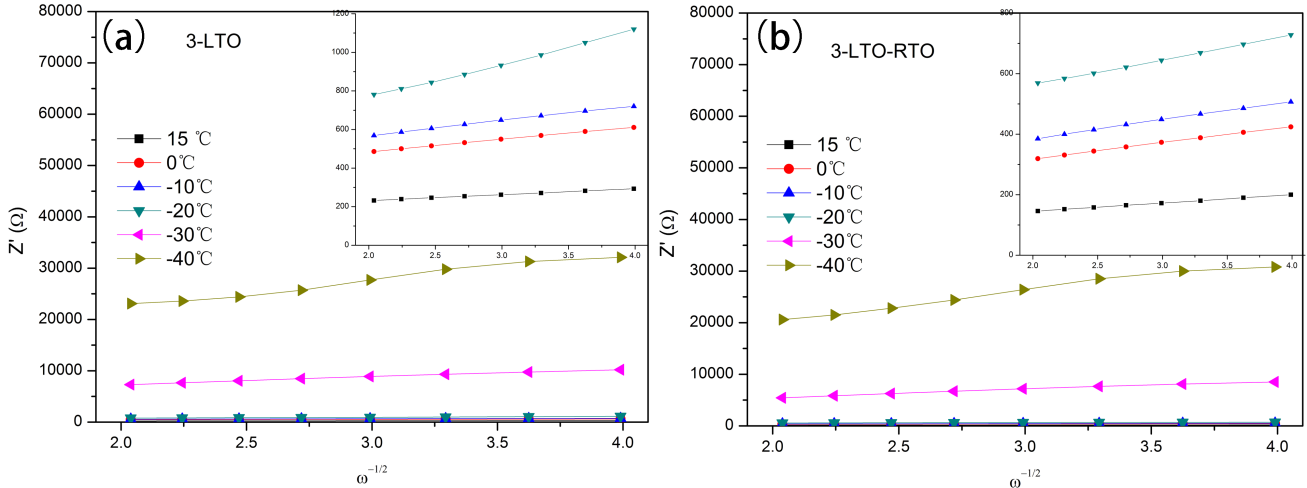


**Figure S3** Z'~$\omega^{-1/2}$ plots of LTO electrode (a) and LTO-RTO electrode at different temperature (b).


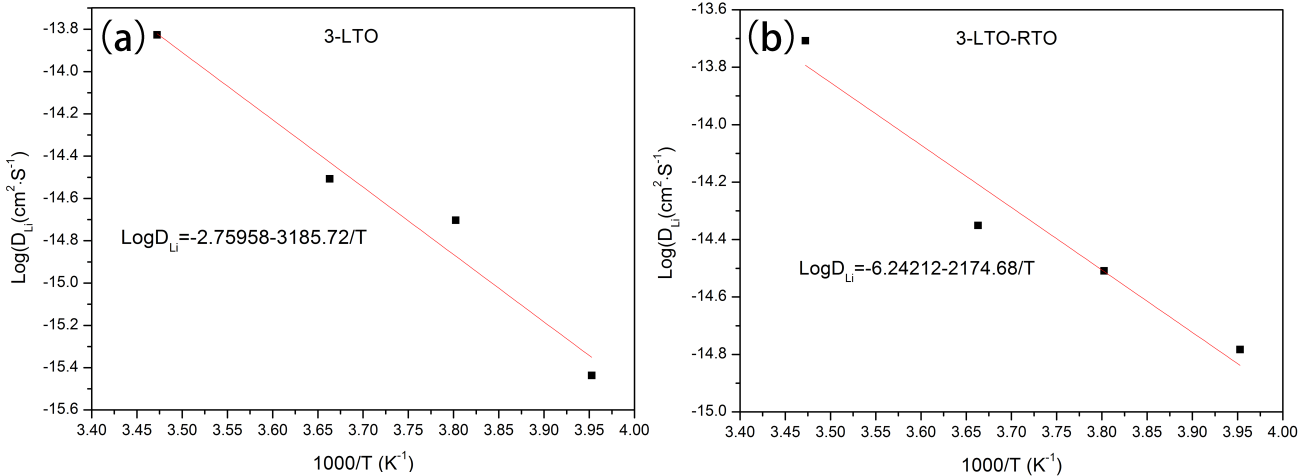


**Figure S4** Arrhenius Plots of the D_Li_~1000/T of LTO (a) and LTO-RTO (b).

**S1. The calculation of D_Li_**

The D_Li_ was calculated using the following equation:

$Z^{'}=R_{s}+R_{\mathrm{sei}}+R_{\mathrm{ct}}+\sigma\omega^{-1/2}$ (1)

$D_{\mathrm{Li}}=\frac{R^{2}T^{2}}{2A^{2}n^{4}F^{4}C_{\mathrm{Li}}^{2}\sigma^{2}}$ (2)

Where Z' is the real part of impedance, R is the gas constants, T is the absolute temperature, A is the surface area of the electrode, n is the number of electrons transferred in half-reaction for the redox couple, F is the Faraday constant, C_Li_ is the concentration of lithium ion in solid, σ is the Warburg impedance coefficient which is closely related to the value of Z'.

**S2. The calculation of E_a_**

The activation energy (E_a_) was calculated using the following equation:

$\mathrm{Log}D_{\mathrm{Li}}=\frac{{-E}_{a}}{\mathrm{RT}}+C$ (3)

$Ea=-Rkln10$ (4)

Where E_a_ is the activation energy, C is the constant and k is the slope of D_Li_~1000/T plots (Figure S4 ). Because the values of D_Li_ at -30 °C and -40 °C are big difference with the values of D_Li_ at 15 °C, 0 °C, -10 °C and -20 °C. Therefore we give up the points of -30 °C and -40 °C to calculate E_a_ based the lithium ion diffusion coefficient.
